# The Injectable Contraceptive Medroxyprogesterone Acetate Attenuates *Mycobacterium tuberculosis–*Specific Host Immunity Through the Glucocorticoid Receptor

**DOI:** 10.1093/infdis/jiy657

**Published:** 2018-11-19

**Authors:** Michele Tomasicchio, Malika Davids, Anil Pooran, Grant Theron, Liezel Smith, Lynn Semple, Richard Meldau, Janet Patricia Hapgood, Keertan Dheda

**Affiliations:** 1 Centre for Lung Infection and Immunity, Division of Pulmonology and UCT Lung Institute, Department of Medicine, University of Cape Town; 2 Department of Science and Technology, National Research Foundation Centre of Excellence for Biomedical Tuberculosis Research, South African Medical Research Council Centre for Tuberculosis Research, Division of Molecular Biology and Human Genetics, Faculty of Health Sciences, Stellenbosch University; 3 Department of Molecular and Cell Biology, University of Cape Town, South Africa; 4 Institute of Infectious Diseases and Molecular Medicine, University of Cape Town, South Africa; 5 Faculty of Infectious and Tropical Diseases, London School of Hygiene and Tropical Medicine, London, UK

**Keywords:** injectable contraceptive, medroxyprogesterone acetate, norethisterone, tuberculosis, pathogenesis

## Abstract

**Background:**

The effects of the widely used progestin-only injectable contraceptives, medroxyprogesterone acetate (MPA) and norethisterone acetate (NET-A), on host susceptibility to *Mycobacterium tuberculosis* (*Mtb*) are unknown.

**Methods:**

We recruited human immunodeficiency virus–uninfected females, not taking any contraceptives, from Cape Town, South Africa, to evaluate the effect of MPA, NET-A, and dexamethasone on *Mtb* containment in monocyte-derived macrophages co-incubated with purified protein derivative (PPD)–driven peripheral blood–derived effector cells.

**Results:**

MPA (*P* < .005) and dexamethasone (*P* < .01), but not NET-A, significantly attenuated *Mtb* containment in *Mtb*-infected macrophages co-cultured with PPD-driven effector cells at physiologically relevant concentrations and in a dose-dependent manner. Antagonizing the glucocorticoid receptor with mifepristone (RU486) abrogated the reduction in *Mtb* containment. In PPD-stimulated peripheral blood mononuclear cells, MPA and dexamethasone, but not NET-A, upregulated (median [interquartile range]) regulatory T cells (5.3% [3.1%–18.2%]; *P* < .05), reduced CD4^+^ T-cell interferon-γ (21% [0.5%–28%]; *P* < .05) and granzyme B production (12.6% [7%–13.5%]; *P* < .05), and reduced CD8^+^ perforin activity (2.2% [0.1%–7%]; *P* < .05). RU486 reversed regulatory T-cell up-regulation and the inhibitory effect on Th1 and granzyme/perforin-related pathways.

**Conclusions:**

MPA, but not NET-A, subverts mycobacterial containment in vitro and downregulates pathways associated with protective CD8^+^- and CD4^+^-related host immunity via the glucocorticoid receptor. These data potentially inform the selection and use of injectable contraceptives in tuberculosis-endemic countries.

## Teaser

Injectable contraceptive usage is high in tuberculosis (TB)–endemic countries, and their effect on TB pathogenesis in humans has not been investigated. At physiological concentrations, medroxyprogesterone acetate, but not norethisterone acetate, attenuates *Mycobacterium tuberculosis* containment and antagonizes pathways associated with protective host immunity through glucocorticoid receptor–mediated mechanisms.

Tuberculosis (TB) is the biggest infectious disease killer globally and remains out of control in several regions of the world [1]. It has been estimated that in 2016, 10.4 million people were living with the disease worldwide and 1.3 million died [2]. The disease pathogenesis remains poorly understood. There are several drivers of TB, including poverty, poor nutrition, human immunodeficiency virus type 1 (HIV-1) co-infection, exposure to biomass fuel combustion, smoking, diabetes, and use of glucocorticoids, among others [1]. However, one widely used and common exposure that has been poorly studied as a potential driver of TB is the use of injectable contraceptives.

The injectable progestin-only contraceptives medroxyprogesterone acetate (MPA) (Depo-Provera; administered every 3 months) and norethisterone enanthate or norethisterone acetate (NET-EN and NET-A, respectively; administered every 2 months), are extensively used in Sub-Saharan Africa (16.5 million users in 2015), where infectious diseases such as *Mycobacterium tuberculosis* (*Mtb*) are endemic [3, 4] and TB is the highest cause of maternal mortality [5]. For example, in KwaZulu-Natal, South Africa, up to 82% of women in rural settings are on an injectable progestin-only contraceptive compared with 15% on oral contraceptives [6]. Even though MPA and NET-A are designed to act via the progesterone receptor (PR), MPA, unlike NET-A, acts as an agonist for the glucocorticoid receptor (GR) [7–10], the activation of which is a well-known risk factor and trigger for TB reactivation [11, 12]. Indeed, the GR regulates a number of genes that are important for TB-related immunity (reviewed in [13]). These considerations are particularly relevant in South Africa, where injectable contraceptive usage is high and TB is widespread.

A recent meta-analysis of clinical observational data suggested that MPA is associated with a significant 40% increase in risk for HIV-1 acquisition, whereas no such association has been found with limited data for NET-A [14]. There is evidence for several possible mechanisms for this effect of MPA, including inhibition of immune function [4] and increased HIV-1 replication in target cells [15]. However, very little is known regarding how hormonal contraceptives affect *Mtb* pathogenesis. In mice, MPA increases *Mtb* bacterial load in the lung [16], but whether a similar effect occurs in humans remains unknown. This is an important question as, at population level, it could represent a major modifiable risk factor that could impact TB control in endemic countries. Thus, we sought to determine whether MPA and/or NET-A diminishes the ability of effector cells to contain *Mtb* in vitro, whether they modulate pathways associated with protective host immunity against TB, and whether these effects are regulated via the GR.

## METHODS

### Study Site and Population

We recruited healthy HIV-1-uninfected contraceptive-free women (n = 11) from Cape Town, South Africa. Patients who were recruited to the study had not used a contraceptive for ≥3 months prior to recruitment. Written informed consent was obtained from all participants. Approximately 100 mL of blood was obtained from each volunteer by a phlebotomist. Ethical approval was obtained from the Research Ethics Committee at the University of Cape Town, South Africa (307/2014).

### Isolation and Culture of Peripheral Blood Mononuclear Cells

Peripheral blood mononuclear cells (PBMCs) were isolated by density centrifugation using Leucosep tubes (Greiner) and Histopaque-1077 (Sigma), according to the manufacturer’s specifications. Cells were cultured in Roswell Park Memorial Institute medium (RPMI) containing 10% human A/B serum (Western Province blood transfusion services, South Africa), 100 IU penicillin/streptomycin, 2 mM l-glutamine, 25 mM HEPES, and 0.1 mg/mL sodium pyruvate (R-10).

### Mycobacterial Containment Assays

The mycobacterial containment assays were performed as described previously [17]. In brief, effector cells were generated by incubating PBMCs (2 × 10^5^ cells/well) in 96-well round-bottom tissue culture plates (Greiner) with or without or in combination with 12 µg/mL purified protein derivative (PPD) (Statens Serum Institut), dexamethasone, MPA, and NET-A (which is metabolized to NET) (Sigma) at concentrations of 10 nM, 50 nM, 100 nM, and 1 µM for 3 days at 37°C. The stock concentrations of dexamethasone, MPA, NET-A, and RU486 (mifepristone) were prepared in 100% ethanol. These compounds are soluble in the growth medium at concentrations ≤1 µM. One-half volume of fresh medium was added containing 10 U/mL interleukin (IL-) 2 (Roche), and the cells were incubated for an additional 4 days at 37°C. IL-2 was added to support the viability and proliferation of the PBMCs. Dexamethasone, a synthetic GR agonist, was included in the study as a control to determine the maximal GR-dependent response in both the containment and flow cytometry assays. For the antagonist studies, 100 nM of dexamethasone, MPA, or NET-A was used in conjunction with 1 µM of the GR and progesterone receptor (PR) antagonist RU486 (Sigma).

In parallel with deriving the effector cells, monocyte-derived macrophages (MDMs; 2 × 10^6^ cells/well) were generated and infected with a multiplicity of infection (MOI) of one *Mtb* H37*Rv* to one MDM (MOI = 1:1). After 18 hours of incubation at 37°C, the cells were washed to remove extracellular *Mtb*. The effector cells were co-cultured with *Mtb*-infected macrophages and the cells were incubated at 37°C for 24 hours. The cells were then washed, and lysed in water, and various dilutions were plated onto Middlebrook 7H10 agar (Merck) supplemented with 10% oleic albumin dextrose catalase (OADC; Becton Dickinson) supplement. After 10–14 days of incubation at 37°C, colony-forming units (CFUs) were counted and expressed as CFU/mL. The percentage (%) mycobacterial containment was calculated relative to the reference control (*Mtb*-infected MDMs only):

$$\text{1}00–\left\{\frac{\text{Experimental condition }\left(\text{CFU /mL}\right)}{\text{Reference control }\left(\text{CFU }/\text{mL}\right)}\right\}\times 100=\%\;M.tb\text{ containment}$$

### Flow Cytometric Determination of Regulatory T-Cell Expansion, Interferon-γ Expression, and Cytotoxic T-Lymphocyte Induction In Vitro

PBMCs (2 × 10^6^ cells) were treated with 12 µg/mL PPD, 100 nM dexamethasone, MPA, NET-A, and/or 1 µM RU486 for 7 days at 37°C. After 7 days, cells were stained with antibodies against CD3, CD4, CD8, CD25, FOXP3, interferon gamma (IFN-γ) (Biolegend), granzyme B, and perforin (Becton Dickinson).

Approximately 1 × 10^5^ cells were acquired using a LSRII Flow Cytometer (Becton Dickinson), and the data were analyzed using FloJo software version 10.1 (TreeStar). Dead cells were excluded from the scatterplots prior to analysis, and negative gates were set using fluorescence minus one controls. Only the single cellular population was analyzed.

### Statistical Analysis

Data were analyzed for statistical significance by one-way analysis of variance with Dunnett post test or Wilcoxon signed-rank paired *t* test using GraphPad Prism software version 6.0. For dose-response analysis, a nonparametric statistical trend test was performed across the concentration range for each compound, using the Wilcoxon signed-rank paired *t* test, as further extended by Cuzick [18], using Stata version 13 software.

## RESULTS

### MPA, but Not NET-A, Decreases Peripheral Effector-Mediated *Mtb* Containment In Vitro in a Dose-Dependent Fashion

To determine if MPA affects *Mtb* pathogenesis, we used a mycobacterial containment assay. In brief, PBMCs were incubated with or without or in combination with 12 µg/mL PPD, 100 nM dexamethasone, MPA, and NET-A for 7 days at 37°C to generate the effector cells. The effector cells were co-cultured with *Mtb*-infected MDMs for 24 hours at 37°C, and the number of CFUs/mL was determined by counting colonies on Middlebrook 7H10 agar containing OADC supplement.

**Figure 1.**
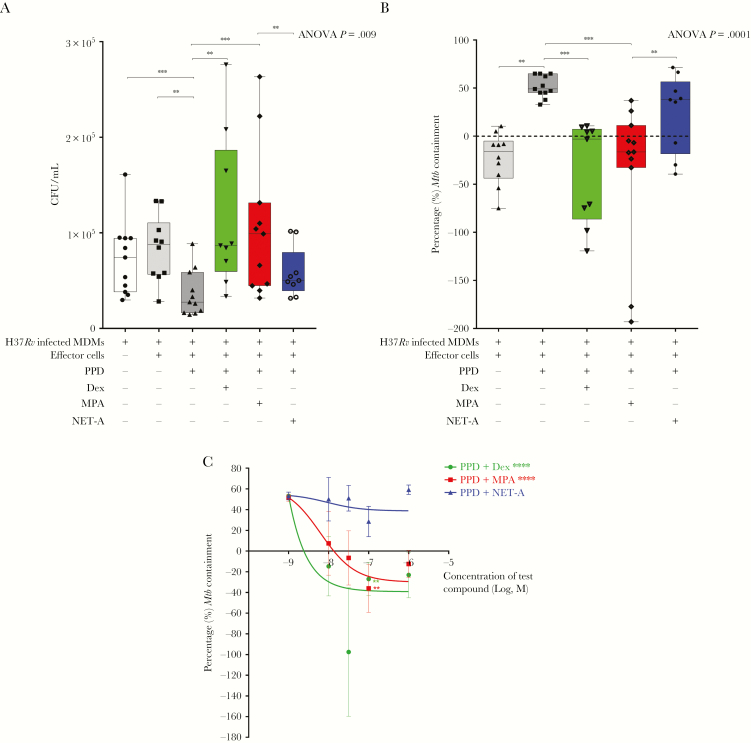
Medroxyprogesterone acetate (MPA), but not norethisterone acetate (NET-A), decreases peripheral effector cell–mediated *Mycobacterium tuberculosis* (*Mtb*) containment in vitro. *Mtb*-infected monocyte-derived macrophages (MDMs) were incubated alone (n = 11) or co-cultured with peripheral blood mononuclear cells that were incubated without (n = 10) or with (n = 11) 12 µg/mL purified protein derivative (PPD) alone and in parallel with physiologically representative concentrations of dexamethasone (n = 9), MPA (n = 11), or NET-A (n = 9). *A*, The number of colony-forming units/mL was determined by counting colonies on Middlebrook H9/OADC agar. *B*, Percentage of mycobacterial containment in the presence of 12 µg/mL PPD alone or in combination with 100 nM dexamethasone, MPA, or NET-A (error bars represent median and interquartile range). The percentage of containment was calculated relative to the reference control (*Mtb*-infected MDMs only); the dotted line represents relative levels of containment by the reference control (*Mtb*-infected MDMs only, no *Mtb* containment). *C*, Dose-dependent levels of percentage of mycobacterial containment in the presence of PPD and increasing concentrations of dexamethasone, MPA, and NET-A (a figure greater than –100 indicates proliferation of the organism; error bars represent mean and standard deviation). A nonlinear fit of the dose response was performed for MPA and dexamethasone. Data were analyzed for statistical significance by one-way analysis of variance withDunnett post test or Wilcoxon signed-rank paired *t* test, where **, *** and **** indicate *P* < .01, *P* < .005 and *P* < .0001, respectively. Statistical trend analysis for each dose response was performed by the Wilcoxon signed-rank paired *t* test, as further extended by Cuzick [18], and showed a significant trend only for dexamethasone (*P* < .0001) and MPA (*P* < .0001). Abbreviations: ANOVA, analysis of variance; CFU, colony-forming units; Dex, dexamethasone; MDM, monocyte-derived macrophage; MPA, medroxyprogesterone acetate; *Mtb*, *Mycobacterium tuberculosis*; NET-A, norethisterone acetate; PPD, purified protein derivative.

The absolute CFUs showed a significant increase (to similar levels as *Mtb*-infected MDMs incubated in the absence of PBMCs [MDM-only control: 7.4 × 10^4^ CFU/mL] or in the presence of PBMCs that were not stimulated with PPD [8.8 × 10^4^ CFU/mL]) when *Mtb*-infected MDMs were co-incubated with PBMCs that were stimulated with PPD and MPA (1 × 10^5^ CFU/mL; *P* < .005) or PPD and dexamethasone (8.7 × 10^4^ CFU/mL; *P* < .01) compared to PPD and NET-A (5 × 10^4^ CFU/mL) or PPD only (2.8 × 10^4^ CFU/mL) (Figure 1A).

We further expressed the data as percentage of *Mtb* containment, which is defined as the percentage change in CFU/mL relative to the MDM-only control (Figure 1B). The control thus represents 0% *Mtb* containment or 100% survival. Similarly, the data showed that PBMCs incubated with PPD and dexamethasone (*P* < .01) or PPD and MPA (*P* < .005) significantly decreased the percentage of *Mtb* containment compared with PBMCs stimulated with PPD alone (Figure 1B). The MPA-mediated (*P* < .01) decrease in percentage of *Mtb* containment was not observed with PBMCs stimulated with PPD and NET-A. The reduction in *Mtb* containment cannot be explained by dexamethasone or MPA reducing the viability of the effector cells more than NET-A, because at the time of co-culture with the *Mtb*-infected MDMs, the levels of effector cells that were alive were similar for all the treatments as assessed by Trypan blue staining (data not shown).

The synthetic GR agonist dexamethasone, and MPA, unlike NET-A, dose-dependently decreased PPD effector-mediated *Mtb* containment within MDMs in vitro (Figure 1C). The levels of dose-dependent containment observed with dexamethasone were higher than MPA, suggesting that MPA was acting as a partial agonist for the GR. Importantly, MPA reduced PPD-mediated effector containment of *Mtb* at physiological concentrations between 10 nM and 100 nM (*P* < .01). This reduction in containment was not observed with NET-A even at concentrations as high as 1 µM. Complete abolishment of *Mtb* containment to similar levels of the reference control were observed at MPA concentrations between 10 nM and 1 µM, with the highest loss of containment observed at 100 nM (Figure 1C). Statistically significant trends [18] were observed for dexamethasone (*P* < .0001) and MPA (*P* < .0001), but not for NET-A, showing decreased *Mtb* containment with increasing concentrations of compound (Figure 1C).

### MPA Reduces PPD-Mediated Effector *Mtb* Containment Through a Mechanism Involving the GR In Vitro

**Figure 2.**
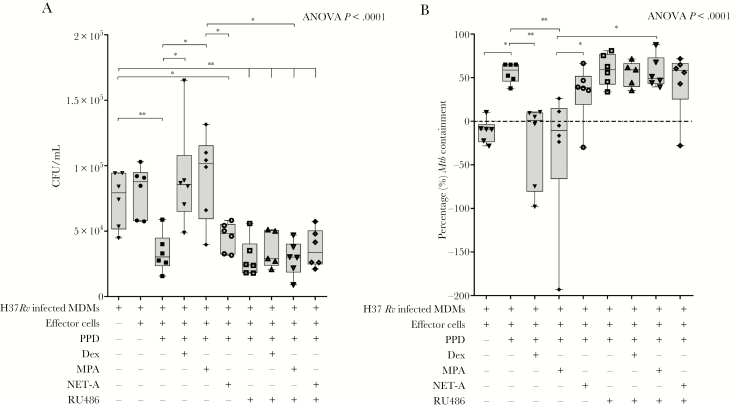
Medroxyprogesterone acetate (MPA) decreases peripheral effector *Mycobacterium tuberculosis* (*Mtb*) containment through the glucocorticoid receptor (GR). *Mtb*-infected monocyte-derived macrophages (MDMs) were cultured alone (n = 6) or were co-cultured with untreated effector cells (n = 6) or effector cells treated with 12 µg/mL purified protein derivative (PPD) alone (n = 6); PPD and 100 nM dexamethasone (n = 6); PPD and 100 nM MPA (n = 6); PPD and 100 nM norethisterone acetate (NET-A) (n = 6); PPD and 1 µM RU486 (a GR antagonist) (n = 6); PPD, dexamethasone, and RU486 (n = 5); PPD, MPA, and RU486 (n = 6); or PPD, NET-A, and RU486 (n = 6). The relative number of colony-forming units/mL (*A*) was determined as indicated previously. The percentage of mycobacterial containment (*B*) was determined as indicated previously to the reference control (*Mtb*-infected MDMs only). Error bars represent median and interquartile range. Data were analyzed for statistical significance by one-way analysis of variance with Dunnett post test or Wilcoxon signed-rank paired *t* test, where * and ** indicate *P* < .05 and *P* < .01, respectively. Abbreviations: ANOVA, analysis of variance; CFU, colony-forming units; Dex, dexamethasone; MDM, monocyte-derived macrophages; MPA, medroxyprogesterone acetate; *Mtb*, *Mycobacterium tuberculosis*; NET-A, norethisterone acetate; PPD, purified protein derivative.

Having shown that MPA can reduce PPD-mediated effector *Mtb* containment in a dose-dependent manner like the synthetic GR agonist dexamethasone, we wanted to determine if the reduction in *Mtb* containment involved the GR. In brief, effector cells were generated as indicated above with or without or in combination with 12 µg/mL PPD, 100 nM dexamethasone, MPA, NET-A, and/or the GR antagonist RU486 (1 µM). The effector cells were co-cultured with *Mtb*-infected MDMs and CFUs/mL were counted as indicated in the methods. As we observed previously, the absolute number of CFUs (to similar levels as *Mtb*-infected MDMs incubated in the absence of effector cells [8.8 × 10^4^ CFU/mL]) were significantly higher when *Mtb*-infected MDMs were co-cultured with PPD and dexamethasone (8.6 × 10^4^ CFU/mL; *P* < .05) or PPD and MPA (1.02 × 10^5^ CFU/mL; *P* < .05; but not PPD and NET-A [48 CFU/mL]) stimulated effector cells compared to when effector cells were stimulated with PPD alone (3 × 10^4^ CFU/mL; Figure 2A). Importantly, when the *Mtb*-infected MDMs were co-cultured with PPD, dexamethasone, and RU486 (a GR agonist; 2.9 × 10^4^ CFU/mL) or PPD, MPA, and RU486 (3 × 10^4^ CFU/mL), the number of CFUs were similar to when effector cells were stimulated with PPD only (3 × 10^4^ CFU/mL), indicating that MPA and dexamethasone were reducing PPD-mediated effector control of *Mtb* through a GR mechanism in vitro (Figure 2A).

As expected, when we represented the data as percentage of *Mtb* containment (as described previously), we observed that both dexamethasone (*P* < .01) and MPA (*P* < .01), but not NET-A, decreased PPD effector-mediated percentage of *Mtb* containment (Figure 2B). In the presence of RU486, the MPA- (*P* < .05) and dexamethasone-mediated decrease in percentage containment was reversed (Figure 2B). The data presented here indicate that the reduced levels of containment observed with MPA involve a GR mechanism.

### MPA, but Not NET-A, Increases Regulatory T-Cell Expansion In Vitro

**Figure 3.**
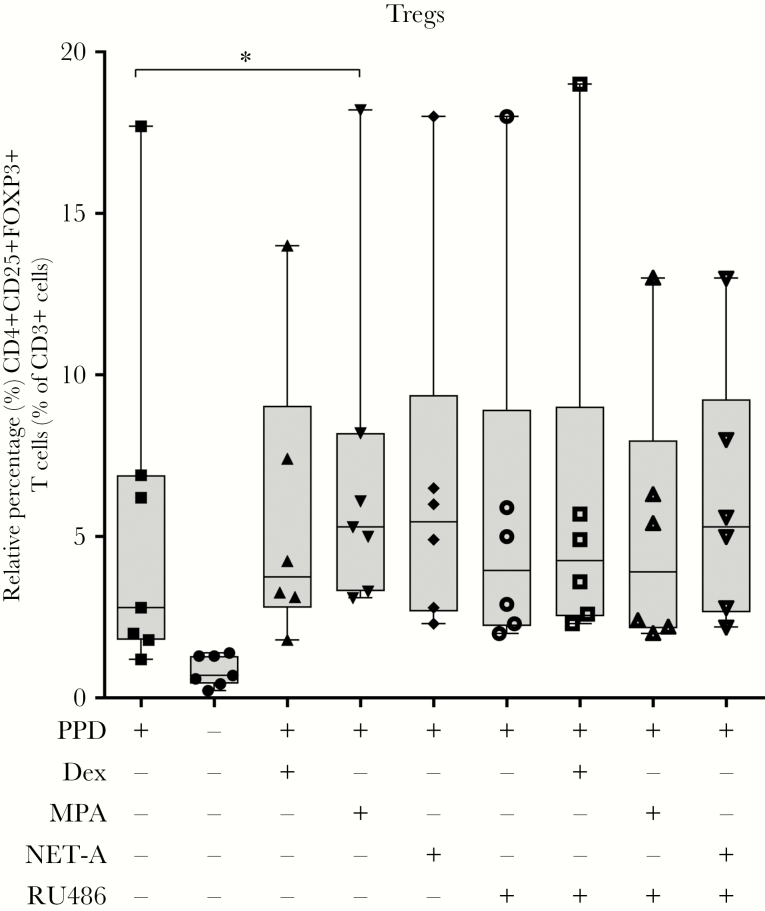
Medroxyprogesterone acetate (MPA), but not norethisterone acetate (NET-A), increases regulatory T-cell (Treg) expansion in vitro, and this is attenuated by RU486. Effector cells were generated by incubating peripheral blood mononuclear cells with (n = 7) or without (n = 7) 12 µg/mL purified protein derivative (PPD) or in combination with 100 nM dexamethasone (n = 6), MPA (n = 7), NET-A (n = 6), and/or 1 µM RU486 (n = 6) for 7 days at 37°C. The levels of Tregs were determined by flow cytometry. Tregs were classified as CD4^+^, CD25^+^, and FOXP3^+^. Dead cells were excluded from the scatterplots prior to analysis, and negative gates were set using fluorescence minus one controls. Only the single cellular populations were analyzed. Error bars represent median and interquartile range. Data were analyzed for statistical significance by one-way analysis of variance with Dunnett post test, where * indicates *P* < .05. Abbreviations: Dex, dexamethasone; MPA, medroxyprogesterone acetate; NET-A, norethisterone acetate; PPD, purified protein derivative; Treg, regulatory T cell.

The data presented thus far indicate that MPA, but not NET-A, reduces *Mtb* containment through the GR. Next, we wanted to determine if the reduction in containment was as a result of immune dysfunction in vitro. PPD-generated effector cells in the presence of the various contraceptives were evaluated for the expansion of regulatory T-cells (Tregs) by flow cytometry. Figure 3 shows that in the presence of PPD and MPA (median, 5.3%; interquartile range [IQR], 3.1%–18.2%), the levels of Tregs were upregulated approximately 2-fold above cells treated with PPD only (median, 2.8%; IQR, 3.1%–18.2%; *P* < .05). No increase in MPA-mediated Treg expansion was observed in the presence of the GR antagonist RU486, and no increase was observed when the cells were treated with PPD, dexamethasone, NET-A, and/or RU486.

### MPA, but Not NET-A, Downregulates PPD-Mediated IFN-γ Expression and Cytotoxic T-Lymphocyte Induction in T Cells Involving the GR

**Figure 4.**
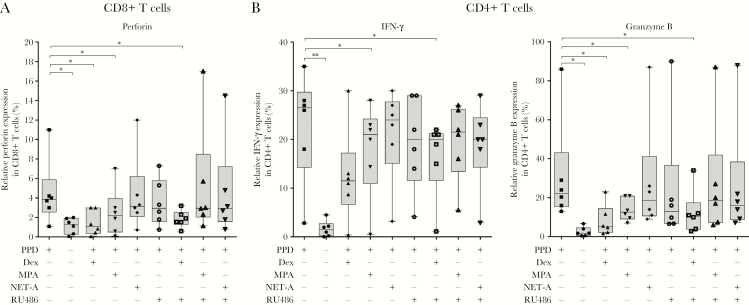
Medroxyprogesterone acetate (MPA), but not norethisterone acetate (NET-A), reduces interferon gamma (IFN-γ) expression and cytotoxic T-lymphocyte (CTL) induction in CD8^+^ and CD4^+^ T cells. Peripheral blood mononuclear cells were treated with (n = 6) or without (n = 6) 12 µg/mL purified protein derivative (PPD) or in combination with 100 nM dexamethasone (n = 6), MPA (n = 6), NET-A (n = 6), and/or 1 µM RU486 (n = 6) for 7 days at 37°C. IFN-γ expression and CTL induction were assessed in the CD8^+^ and CD4^+^ T cells using flow cytometry. Error bars represent median and interquartile range. Data were analyzed for statistical significance by one-way analysis of variance with Dunnett post test, where * and ** indicate *P* < .05 and *P* < .01, respectively. Abbreviations: Dex, dexamethasone; IFN-γ, interferon gamma; MPA, medroxyprogesterone acetate; NET-A, norethisterone acetate; PPD, purified protein derivative.

Next, we wanted to determine the effect of MPA on IFN-γ expression and cytotoxic T-lymphocyte (CTL) induction, in CD4^+^ and CD8^+^ T cells in vitro (Figure 4). MPA (median, 2.2%; IQR, 0.1%–7%; *P* < .05), but not NET-A (median, 3.2%; IQR, 0.07%–12%), had the ability to reduce PPD-induced perforin expression (an important cytolytic protein; median, 3.9%; IQR, 1.08%–11%) in CD8^+^ T cells. This effect was abrogated in the presence of RU486 (median, 3%; IQR, 1.1%–17%), indicating that the GR was involved. Similarly, dexamethasone (median, 1.1%; IQR, 0.1%–3%; *P* < .05) also reduced perforin induction by PPD (median, 3.9%; IQR, 1.08%–11%) in CD8^+^ T cells, but surprisingly this effect was not reversed with RU486 (median, 1.7%; IQR, 0.6%–3.2%), suggesting that it occurs through a GR-independent mechanism (Figure 4A). In contrast, MPA had no effect on IFN-γ and granzyme B (a marker for cytotoxicity) expression in the CD8^+^ T cells (Supplementary Figure 1). Our data further showed that MPA had no effect on the expression of tumor necrosis factor alpha (TNF-α), IL-10, IL-17, CD69, or IL-13 in the CD8^+^ T cells (Supplementary Figure 1).

We further show that MPA (median, 21%; IQR, 0.5%–28%; *P* < .05), but not NET-A (median, 24%; IQR, 3.2%–30%) reduced PPD-induced (median, 27%; IQR, 2.8%–35%) expression of IFN-γ in CD4^+^ T cells (Figure 4B). A similar trend was observed with dexamethasone (median, 11%; IQR, 0.4%–30%). As was observed in the CD8^+^ T cells, both dexamethasone (median, 5.1%; IQR, 1.6%–11.6%; *P* < .05) and MPA (median, 12%; IQR, 7%–21%; *P* < .05), but not NET-A (median, 18.5%; IQR, 9%–87%), repressed PPD-induced (median, 22.2%; IQR, 13%–86%) granzyme B expression in the CD4^+^ T cells (Figure 4B). This repression with MPA was reversed with RU486 (median, 18.5%; IQR, 6%–87%) in the CD4^+^ T cells, indicative of a GR-mediated mechanism. We observed no differences in the expression of perforin, GATA-3, IL-10, TNF-α, CD69, T-box protein expressed in T cells (Tbet), IL-17, or IL-13 in the CD4^+^ T cells stimulated with PPD and dexamethasone, PPD and MPA or PPD and NET-A (Supplementary Figure 2).

## DISCUSSION

We show for the first time that MPA at physiological concentrations, but not NET-A, decreases T-cell effector–mediated containment of *Mtb* through a mechanism involving the GR in human PBMCs. Furthermore, the decrease in containment was associated with increased Treg expansion, decreased IFN-γ expression, and reduced CTL induction in vitro.

The key finding was that MPA, but not NET-A, decreased *Mtb* containment in vitro (up to 2.6-fold) in a dose-dependent manner. The relative dose-dependent decrease with MPA was lower than that produced by dexamethasone, suggesting that MPA was acting as a partial agonist for the GR, which is consistent with the literature [8, 19, 20]. Remarkably, MPA appeared to decrease the levels of *Mtb* containment at concentrations as low as 10 nM. The peak serum concentration (C_max_) of MPA in the peripheral blood of women has been reported to range from 3 to 100 nM [21–25], while NET reaches a peak concentration of 50 nM [26]. While 100 nM is higher than most reports for serum MPA C_max_, given the high interindividual and interstudy variability in reported C_max_ values in women [27], responses to 100 nM are most likely indicative of maximal possible effects in vivo. After 2–3 months, the levels of MPA decreased to between 2.6 and 21 nM, while the levels of NET-A decreased to 13 nM after 2 months [26]. Our results therefore suggest that MPA at physiological concentrations could impact TB pathogenesis and drive *Mtb* replication in the host, thus increasing the risk of acquiring active or latent TB infection.

The findings are broadly concordant with murine data indicating that MPA increased *Mtb* bacterial load in the lungs [16]; however, the immune systems of mice vs humans are different and direct comparisons are misleading. Another study investigated the effects of MPA on *Mycobacterium bovis* bacillus Calmette-Guérin (BCG) in human PBMCs [28]. BCG reduces IL-1α, IL-12p40, IL-10, IL-13, and granulocyte-colony stimulating factor (G-CSF) levels in vitro. However, all BCG strains are missing the region of difference 1 (RD-1) from their genome [29, 30], which expresses key *Mtb* virulence factors [31]. The current study is the first to investigate the effects of injectable contraceptives on *Mtb* pathogenesis, the first to investigate the anti-mycobactericidal effects of injectable contraceptives in human PBMCs, and the first to directly compare the immunopathological effects of 2 different injectable contraceptives.

Our data show that MPA increased Treg expansion in vitro. However, in the presence of the GR antagonist RU486, this increase was not observed, suggesting that the GR was involved. Treg frequency is increased in patients with TB [17] and has been shown to downregulate effector functions of CD8^+^ and CD4^+^ T cells [32], inhibit Th1 effector responses [33], and impair the ability of alveolar and monocyte-derived macrophages to restrict the growth of *Mtb* in the presence of effector cells [17]. In addition, glucocorticoids have been shown to increase Treg expansion in the peripheral blood of asthma patients [34]. Thus, our data indicate that MPA could further suppress protective immune responses to TB, allowing bacterial persistence.

MPA and dexamethasone, but not NET-A, downregulated perforin and granzyme B expression in CD8^+^ and CD4^+^ T cells, respectively, which was reversed with RU486, suggesting that the GR was involved. Others have also shown that glucocorticoids downregulate granzyme B and perforin [35]. Our results show that MPA is acting like a glucocorticoid in this context. It has been documented in human [36] and transgenic mouse models [37] that granule-dependent killing of *Mtb* involving granulysin, granzymes, and perforin are essential to lyse and kill *Mtb*-infected cells. Perforin is essential for pore-mediated delivery of granzyme B into the cell to induce caspase-dependant apoptosis [38], and a robust CTL response is indispensable for human protective host immunity [39]. It is unclear whether the effects of MPA are mediated through CD4^+^ or CD8^+^ T cells. However, we noted changes in IFN-γ and perforin expression, all associated with host protection, in both CD4^+^ and CD8^+^ T cells. While CD4^+^ T cells are incontrovertibly implicated in TB pathogenesis, the role of CD8^+^ T cells is less clear and supported largely by murine rather than direct human data [40, 41].

MPA also appeared to reduce expression of IFN-γ in CD4^+^ T cells, crucial for activation of macrophages to eradicate *Mtb* [42]. Collectively, these data indicate that MPA suppresses CTL-mediated killing of the PPD-activated cells through decreased perforin and granzyme B expression, and Th1 activation in vitro, thus inhibiting effector function and likely bacterial clearance.

We further report that MPA decreased *Mtb* containment through a mechanism involving the GR. It has been previously shown that MPA, but not NET-A, can act as a partial or full agonist for the GR [8, 19, 20], and our data are consistent with previous reports. While MPA could exert its effects via other steroid receptors such as the androgen receptor (AR) and PR [9, 10], we have previously shown that human PBMCs express the estrogen receptor (ER) and mineralocorticoid receptor (MR), but no detectable AR or PR [43]. Furthermore, MPA binds weakly to the MR [44] and does not bind to the ER [45].

There are a number of limitations to the current study. First, the ability of the effector cells to control the bacterium was defined by percentage of containment in this study. Percentage of containment or antimycobacterial activity is difficult to quantify and may involve bacterial killing or induction of a nonreplicating state (we are unable to distinguish between these 2 processes). The containment assay used in this study is a much more powerful, direct, and biologically meaningful outcome measure than inference from the biomarker-specific proxy markers of protection (cytokines, etc) that are often used in human studies but may merely represent epiphenomena rather than causality. Second, in the flow cytometry experiments, we used PPD rather than live *Mtb.* PPD is a *Mtb* lysate that is routinely used to activate and prime effector cells in in vitro studies to reciprocate live *Mtb*. Furthermore, PPD is routinely used in a diagnostic skin test to determine if a patient has been exposed to *Mtb*. The test measures a hypersensitive immune response to PPD. Therefore, the host immune modulatory capacity of PPD vs live *Mtb* would be expected to be similar. All our experiments were performed using PBMCs in vitro, which may not truly be representative of the immune milieu of the lung compartment in vivo. However, using lung cells was not possible due to feasibility, funding, and ethical constraints. It would have been interesting to investigate the causal relationship between Tregs and mycobacterial containment, given that we have previously shown that Tregs subvert mycobacterial containment in humans [17, 46]. However, this was not performed due to funding constraints.

In conclusion, our data indicate that MPA, but not NET-A, attenuates *Mtb* containment and antagonizes pathways associated with protective host immunity through GR-mediated mechanisms. They highlight the potential risks of MPA usage in TB-endemic countries, possibly unlike NET-A. Clinical studies are urgently required to inform policy and delineate the role of MPA in the pathogenesis, acquisition, and dissemination of TB.

## Supplementary Data

Supplementary materials are available at *The Journal of Infectious Diseases* online. Consisting of data provided by the authors to benefit the reader, the posted materials are not copyedited and are the sole responsibility of the authors, so questions or comments should be addressed to the corresponding author.

Supplementary Figure S1

Supplementary Figure S2

Supplementary Material

## Notes

***Acknowledgments.*** The authors thank all of the patients for their participation in this study, and Mohammed Fadul for his input with the statistical analysis.

***Author contributions.*** K. D., M. T., M. D., A. P., G. T., and J. P. H. conceived the idea and designed the experiments. M. T., M. D., A. P., L. S., and R. M. performed the experiments. K. D. and M. T. analyzed the data. K. D., M. T., M. D., A. P., G. T., L. S., and J. P. H. wrote the manuscript.

***Financial support.*** This work was supported by the South African Medical Research Council through its TB and HIV Collaborating Centres Programme with funds received from the National Department of Health.

***Potential conflicts of interest.*** All authors: No reported conflicts of interest. All authors have submitted the ICMJE Form for Disclosure of Potential Conflicts of Interest. Conflicts that the editors consider relevant to the content of the manuscript have been disclosed.
