# Supplementary figures and images for: The Injectable Contraceptive Medroxyprogesterone Acetate Attenuates Mycobacterium tuberculosis–Specific Host Immunity Through the Glucocorticoid Receptor
Source: J Infect Dis. 2018 Nov 19;219(8):1329–37. doi: 10.1093/infdis/jiy657 (PMC6452311; doi:10.1093/infdis/jiy657)

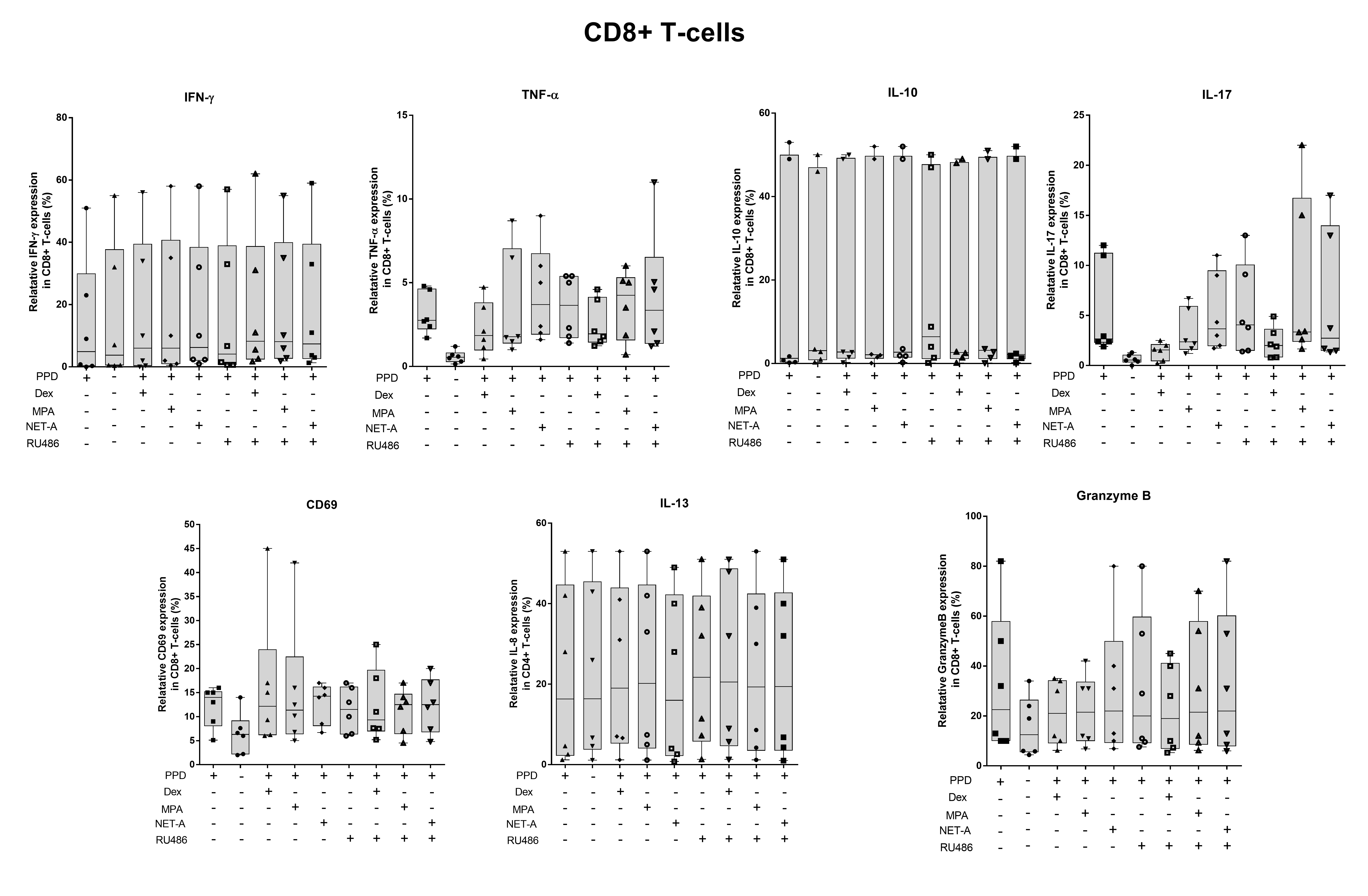

Supplement: Supplementary Figure S1 [file jiy657_suppl_supplementary_figure_s1.png]

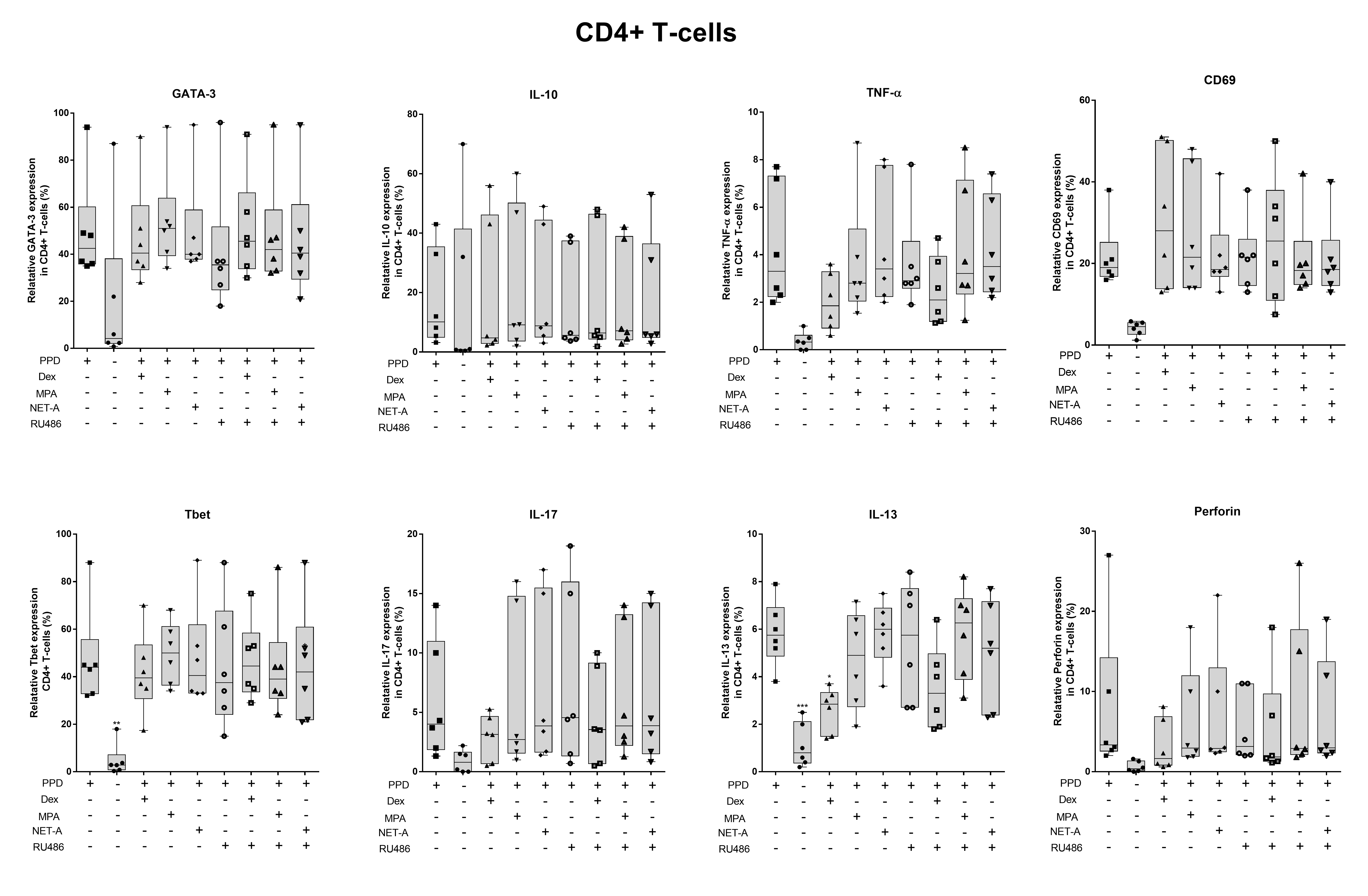

Supplement: Supplementary Figure S2 [file jiy657_suppl_supplementary_figure_s2.png]
