# Supplementary material for: The Injectable Contraceptive Medroxyprogesterone Acetate Attenuates Mycobacterium tuberculosis–Specific Host Immunity Through the Glucocorticoid Receptor
Source: J Infect Dis. 2018 Nov 19;219(8):1329–37. doi: 10.1093/infdis/jiy657 (PMC6452311; doi:10.1093/infdis/jiy657)
Supplement: Supplementary Material [file jiy657_suppl_supplementary_material.docx]

**SUPPLEMENTARY MATERIAL**

The injectable contraceptive medroxyprogesterone acetate attenuates *Mycobacterium tuberculosis*-specific host immunity through the glucocorticoid receptor

Michele Tomasicchio^1^, Malika Davids^1^, Anil Pooran^1^, Grant Theron^2,1^, Liezel Smith^1^, Lynn Semple^1^, Richard Meldau^1^, Janet Patricia Hapgood^3,4^, Keertan Dheda^1,4*^

**Affiliations:**

^1^ Centre for Lung Infection and Immunity, Division of Pulmonology and UCT Lung Institute, Department of Medicine, University of Cape Town, Cape Town, South Africa.

^2^Department of Science and Technology/National Research Foundation Centre of Excellence for Biomedical Tuberculosis Research, South African Medical Research Council Centre for Tuberculosis Research, Division of Molecular Biology and Human Genetics, Faculty of Health Sciences, Stellenbosch University, Cape Town, South Africa.

^3^ Department of Molecular and Cell Biology, University of Cape Town, Cape Town, South Africa.

^4^Institute of Infectious Diseases and Molecular Medicine, University of Cape Town, Cape Town, South Africa.

***Correspondence:** Keertan Dheda, Lung Infection and Immunity Unit, Division of Pulmonology and UCT Lung Institute, Dept of Medicine, University of Cape Town, South Africa.

E-mail: [keertan.dheda@uct.ac.za](mailto:keertan.dheda@uct.ac.za).

Tel: +27 214066509.

Fax: +27 216503824.

**Results**.


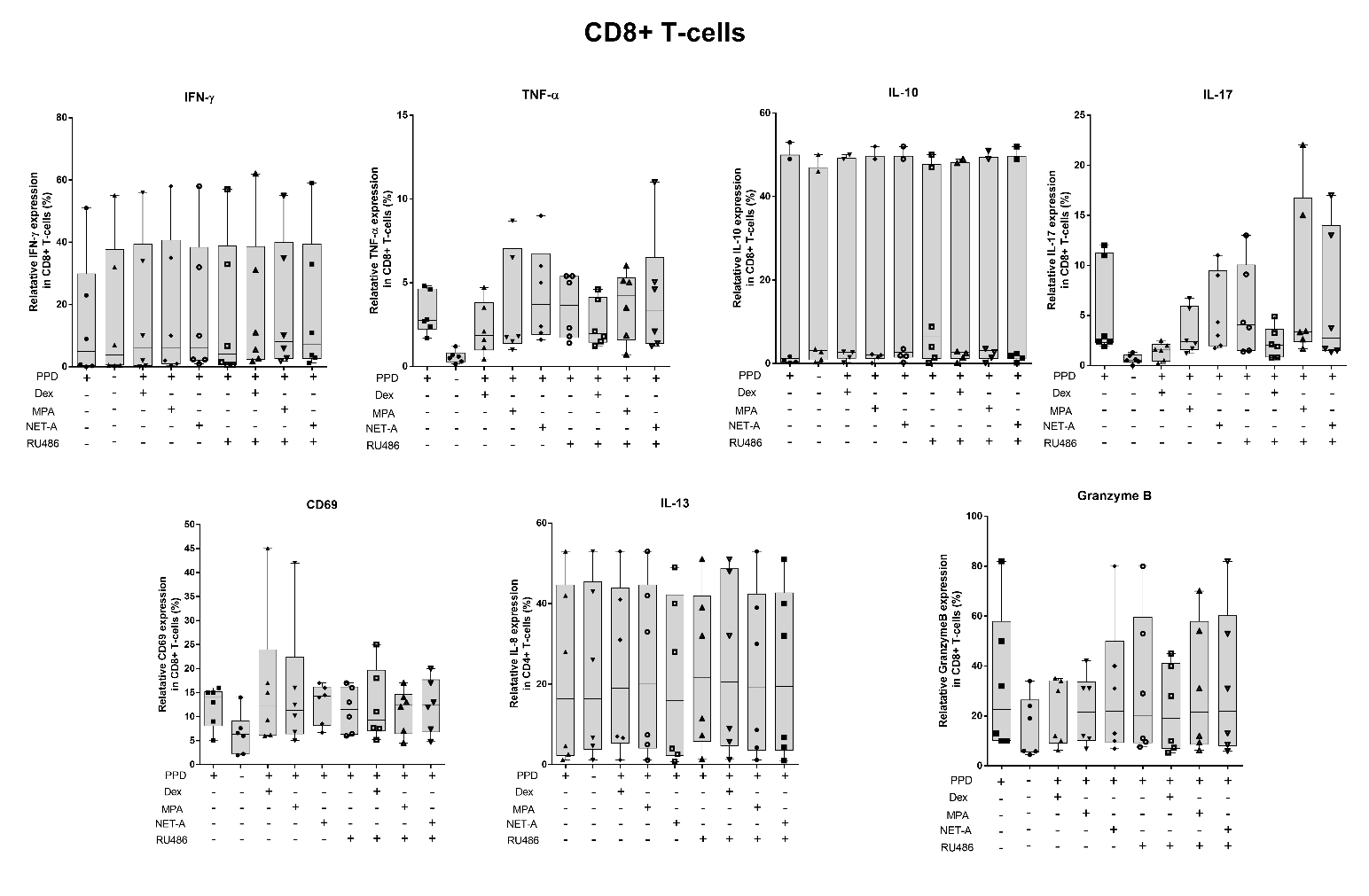


**Figure S1. MPA has no effect on the expression of key cytokines and proteins involved in regulating Th1, Th2 and Th17, CTL-specific immunity in CD8+ T-cells.** PBMCs were treated with or without 12 µg/ml PPD alone or in combination with 100 nM dexamethasone, MPA, NET-A and/or 1 µM RU486 for 7 days at 37°C. Cytokine and protein expression were assessed in the CD8+ T-cells using flow cytometry. The data was analysed as indicated previously and the histogram shows pooled results from six independent experiments and samples from six donors. Error bars represent median interquartile range. Data was analysed for statistical significance by one-way ANOVA with Dunnett’s post-test, where *, **, *** and **** indicate p < 0.05, p < 0.01, p < 0.005 and p < 0.0001, respectively.


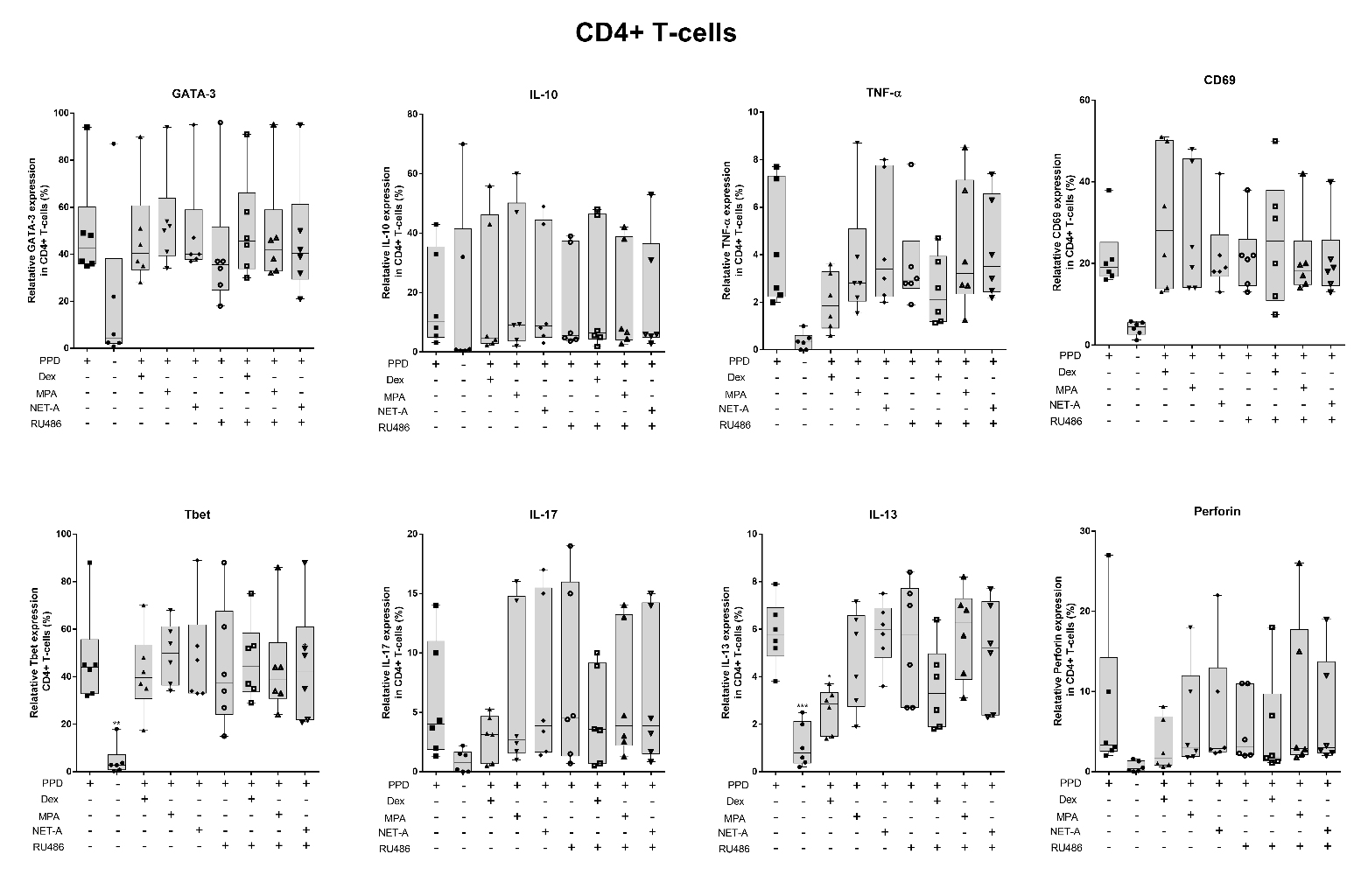


**Figure S2. MPA has no effect on the expression of key cytokines and proteins involved in regulating Th1 and Th2 Th17 and CTL-specific immunity in CD4+ T-cells.** PBMCs were treated with or without 12 µg/ml PPD alone or in combination with 100 nM dexamethasone, MPA, NET-A and/or 1 µM RU486 for 7 days at 37°C. Cytokine and protein expression were assessed in the CD4+ T-cells using flow cytometry. The data was analysed as indicated previously and the histogram shows pooled results from six independent experiments and samples from six donors. Error bars represent median interquartile range. Data was analysed for statistical significance by one-way ANOVA with Dunnett’s post-test, where *, **, *** and **** indicate p < 0.05, p < 0.01, p < 0.005 and p < 0.0001, respectively.
